# Japanese quails (*Coturnix Japonica*) show keel bone damage during the laying period—a radiography study

**DOI:** 10.3389/fphys.2024.1368382

**Published:** 2024-03-06

**Authors:** Lisa Hildebrand, Christoph Gerloff, Birthe Winkler, Beryl Katharina Eusemann, Nicole Kemper, Stefanie Petow

**Affiliations:** ^1^ Institute of Animal Welfare and Animal Husbandry, Friedrich-Loeffler-Institut, Celle, Germany; ^2^ Institute of Animal Hygiene and Public Veterinary Services, Faculty of Veterinary Medicine, Leipzig University, Leipzig, Germany; ^3^ Institute for Animal Hygiene, Animal Welfare and Farm Animal Behaviour, University of Veterinary Medicine Hannover, Hannover, Germany

**Keywords:** keel bone damage, Japanese quails, animal welfare, keel bone fracture, keel bone deviation, radiography, keel bone deformities, ossification

## Abstract

Keel bone damage is an important welfare issue in laying hens and can occur with a high prevalence of up to 100% of hens within one flock. Affected hens suffer from pain. Although multiple factors contribute to the prevalence and severity of keel bone damage, selection for high laying performance appears to play a key role. With up to 300 eggs/year, Japanese quails show a high laying performance, too, and, thus, may also show keel bone damage. However, to our knowledge, there are no scientific results on keel bone damage in Japanese quails to date. Therefore, the aim of this study was to assess whether keel bone fractures and deviations occur in Japanese quails and to obtain more detailed information about the development of their keel bone during the production cycle. A group of 51 female quails were radiographed at 8, 10, 15, 19, and 23 weeks of age. The X-rays were used to detect fractures and deviations and to measure the lateral surface area, length, and radiographic density of the keel bone. In addition, the length of the caudal cartilaginous part of the keel bone was measured to learn more about the progress of ossification. At 23 weeks of age, quails were euthanized and their macerated keel bones assessed for fractures and deviations. Both keel bone deviations and keel bone fractures were detected in the Japanese quails. In the 23rd week of age, 82% of the quails had a deviated keel bone as assessed after maceration. Furthermore, there was a decrease in radiographic density, lateral surface area, and length of the keel bone between weeks of age 8 and 19. This could indicate a general loss of bone substance and/or demineralization of the keel bone. Our study shows that keel bone damage is not only a problem in laying hens but also affects female Japanese quails.

## 1 Introduction

Keel bone damage is one of the most important animal welfare issues in chicken laying hens (Jinman, 2013; Nielsen et al., 2023). The prevalence of fractures can reach up to 100% of hens within one flock (Käppeli et al., 2011; Baur et al., 2020; Thøfner et al., 2021) and affected hens experience pain (Nasr et al., 2012; Nasr et al., 2013; Riber et al., 2018). Furthermore, they are restricted in their mobility and have a lower laying performance (Nasr et al., 2013; Rufener et al., 2019). Thus, keel bone damage has economic effects as well.

The term “Keel bone damage” includes both, keel bone fractures and keel bone deviations. A keel bone deviation is defined as an abnormal shape of the keel bone that is not due to a fracture and varies from the perfect two-dimensional shape of the keel bone (Casey-Trott et al., 2015).

Keel bone fractures are shown as “sharp bends, shearing, and/or fragmented sections of the keel bone” (Casey-Trott et al., 2015). In terms of location, the majority of fractures are found in the caudal third of the keel (Baur et al., 2020; Habig et al., 2021; Kittelsen et al., 2021). Since ossification of the avian sternum proceeds from cranial to caudal, this portion is usually still cartilage at the onset of lay (Wang et al., 2021).

Radiography is a versatile tool to assess keel bone damage. It enables the comparison of different hens as well as longitudinal studies of the same animals (Eusemann et al., 2018a). Besides a high sensitivity and specificity for the detection of fractures, it also offers further possibilities to measure other keel bone parameters. As conducted by Eusemann et al. (2018a), X-ray pictures can be used to measure and calculate the proportion of deviated keel bone area (POD) and thereby quantify the severity of deviations. Another method to quantify keel bone damage on X-ray images is rating it using a tagged visual analog scale as introduced by Rufener et al. (2018) for fractures and Jung et al. (2022) for deviations. Furthermore, Fleming et al. (2000) established a method to assess the bone density of the humerus and tibiotarsus *in vivo* by including an aluminum step wedge on the X-ray picture. This method, which has later been adapted to measure the radiographic density of the keel bone (Eusemann et al., 2020), allows the investigation of the radiographic density at different time points in a longitudinal study. A lower bone density has been linked to more severe keel bone deviations in caged layers and is also associated with pathological fractures through osteoporosis (Dobbs et al., 1999; Habig et al., 2021). In addition, egg-laying hens were found to have a lower radiographic density of the keel bone compared to non-egg-laying hens (Eusemann et al., 2020).

Shortly before the onset of lay, estradiol-17ß, together with androgens, induces the formation of medullary bone (Dacke, 1979; Dacke et al., 1993; Whitehead, 2004). In histologic images, this bone structure can be seen as basophil short spicules extending from the endosteal surface of the cortical and trabecular bone to the medullary cavity (Wilson and Duff 1990; Bloom et al., 1958). This non-structural type of bone serves as a labile calcium source and shows rapid reduction and new formation throughout the egg-laying cycle (Bloom et al., 1941; Bloom et al., 1958; Whitehead, 2004; Kerschnitzki et al., 2014). Loss of structural bone has been linked to the formation of medullary bone by Wilson and Thorp (1998). The authors found a loss of trabecular bone after inducing medullary bone formation in male fowl through estradiol treatment and could also show that a treatment of female fowl with the estrogen receptor modulator Tamoxifen prevented both, formation of medullary bone and loss of trabecular bone. In another study conducted by Dacke et al. (1993), medullary bone recovery during a calcium deficiency was accompanied by a loss of cortical bone. However, there are other studies in which a negative effect of estrogen treatment on cortical bone could not be confirmed (Squire et al., 2017; Eusemann et al., 2022).

Habig et al. (2021) found a higher prevalence of keel bone fractures in high-performing layer lines than in low-performing layer lines. This was also the case within brown layer lines in a study by Eusemann et al. (2018a). A link between keel bone fractures and egg-laying activity is also made by Eusemann et al. (2020). They showed that laying hens prevented from egg-laying through a Deslorelin acetate releasing implant had fewer keel bone fractures than control hens without an implant. This was also the case in hens that additionally received an estrogen releasing implant. Those hens did not lay eggs but built medullary bone. In a study conducted by Kittelsen et al. (2021), the authors found significantly fewer keel bone fractures in red jungle fowl hens compared to White Leghorn hens, a selected laying breed. Red jungle fowl is the non-domesticated species of our laying hens that has never been selected for high laying performance.

This suggests that other species that have been selected for high laying performance may also suffer from keel bone damage. Japanese quails have been domesticated since at least the 12th century (Kovach, 1974) and selected for egg production since 1910 (Yamashina, 1961). As a result, Japanese quails can show a laying performance of 250–300 eggs per year and attain sexual maturity at five to 6 weeks (Yamashina, 1961; Gerken and Mills, 1993). Thus, it is possible that keel bone damage may be an animal welfare problem in Japanese quail hens, too.

The aim of our study was to investigate whether female Japanese quails show keel bone damage and to characterize the keel bone throughout their laying period using radiographic imaging.

## 2 Animals, materials, and methods

### 2.1 Ethical statement

The animal experiment was approved by the competent authority (Lower Saxony State Office for Consumer Protection and Food Safety; no. 33.9-42502-04-13/1096). The competent authority had been informed about certain changes done in the experimental procedure. The quails in the experiment were kept and managed following the general requirements laid down in the German Animal Welfare Act (Tierschutzgesetz, TierSchG) and according to the Directive 2010/63/EU. The animals were daily controlled for health and welfare problems.

### 2.2 Birds and housing conditions

For the experiment, 119 Japanese quails of a layer strain were bred and raised at the Institute of Animal Welfare and Animal Husbandry of the Friedrich-Loeffler-Institut in Celle, Germany. All animals descended from a parent line kept in our facility and hatched on the same day.

After hatching, the quails were moved to a barn compartment of 24 m^2^ where they were kept in a floor housing system littered with sand. The maximum stocking density amounted to five animals per square meter in the first 4 weeks of age The barn compartment was cleaned as required and the sand was replaced every 3 weeks.

A heating lamp was placed above the middle of the area to provide extra warmth for the first 2 weeks and then removed. In the first week, the quails were kept at a compartment temperature of 35°C–38°C. From day 6 to day 18, the temperature decreased continuously until reaching 18°C and then remained constant for the remainder of the experiment.

The pen was equipped with two nipple drinking troughs and two feed dispensers that provided feed *ad libitum*. The animals received quail diets of the Friedrich-Loeffler-Institut. For the first 3 weeks of age, they were fed quail chick feed (11,37 MJ AMEN/kg, 272 g/kg crude protein, 37 g/kg crude fat, 12,0 g/kg Ca, 8.5 g/kg P), from weeks four to six feed for quail pullets (11,84 MJ AMEN/kg, 213 g/kg crude protein, 40 g/kg crude fat, 11,0 g/kg Ca, 7.4 g/kg P). Thereafter, they received a complete feed for laying hens (11.2 M J AMEN/kg, 155 g/kg crude protein, 52,4 g/kg crude fat, 35 g/kg Ca, 5.5 g/kg P).

After hatching, the light duration was reduced from 24 h on the first day to 16 h on the 25th day and then remained constant for the remainder of the experiment.

In the fifth week of age, the male quails, which can be recognized by their feathering at this stage, were captured and removed from the compartment. The remaining 51 female quails were marked with individual wing tags. From there on, the maximum stocking density in the barn was 2.13 animals per square meter. Unfortunately, some of the hens died during the experiment so that at the end, there were 41 hens left and the number of hens differed between examination dates. *Post mortem* dissection revealed a wide range of causes of death including enteritis, salpingitis, pneumonia, dystocia and injuries from cannibalism and accidents. The numbers of hens that were included in the analysis of each parameter at a certain examination date are given in the results section.

### 2.3 Methods

During the experimental period, the quails were investigated five times. The first three dates were set according to the course of the laying period. The first session took place in their eighth week of age as Japanese quails normally start laying between their fifth and seventh weeks of age. The second session was conducted in week 10 when quails reach their maximum of laying performance. In week 15, when the laying performance is still high, the third investigation took place. The last two investigations were performed with an interval of 4 weeks in the 19th and 23rd week of age to ensure the detection of fractures before complete healing. At each of these dates, body weight was recorded and X-rays were taken of all hens. After the last examination at 23 weeks of age, the hens were killed and keel bones were macerated to verify the radiographic findings.

Additionally, a histological analysis of Japanese quail keel bones was conducted to gain more information about the structure and composition of the bone in laying quails, especially about the presence of medullary bone in the keel bone.

#### 2.3.1 Examination of general condition and body weight

At each of the above mentioned dates, the birds were caught in their barn compartment and transported to the examination room in boxes. They were separately unloaded from the box and weighed with a precision scale IP65 (Sartorius, Göttingen, Germany), The general health status was visually assessed, and the footpads were examined to determine the occurrence of pododermatitis before continuing with the X-ray.

#### 2.3.2 Radiographic examination

Radiographs were taken with the X-ray generator WDT Blueline 1040 HF (Wirtschaftsgenossenschaft Deutscher Tierärzte eG, Garbsen, Germany), the digital flat panel detector Thales Piximum 2430 EZ wireless (Thales Electron Devices S.A., Velizy-Villacoublay, France), and a laptop, which was transported in an X-ray case Leonardo DR mini (Oehm and Rehbein GmbH, Rostock, Germany).

For the X-ray, the quails were fixated upside down in a home-built rack. A left latero-lateral radiography was taken with 50.0 kV at 2 mAs. As conducted by Eusemann et al. (2020), a home-built 2 cm * 17.8 cm aluminum step wedge was positioned on the flat panel detector and radiographed with each bird. The 17 steps of the step wedge decreased by 0.25 cm each step and were used to determine the radiographic density of the keel bone on the X-ray image (see below).

#### 2.3.3 Radiographic evaluation

Each X-ray picture was examined for fractures, deviations, radiographic density, total lateral surface area, and length of the keel bone as well as the length of the caudal cartilaginous part of the keel bone (Supplementary Material 1).

**FIGURE 1:**
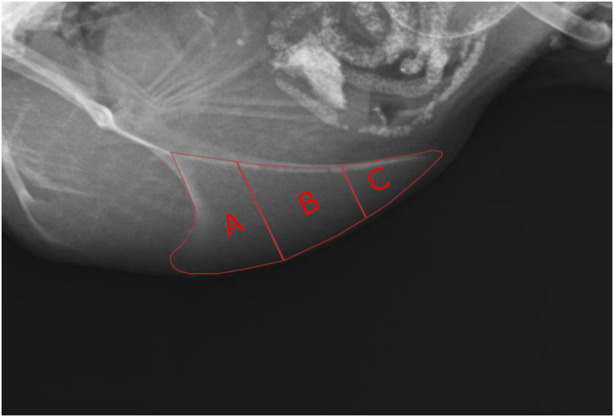
Subdivision of the keel bone into sections for the detection of fractures. **(A)** = cranial section, **(B)** = middle section, **(C)** = caudal section.

To gain more information about the location of the fractures within the keel bone, it was divided into three sections from cranial to caudal as conducted by Baur et al. (2020) excluding the labeling of the apices (Figure 1). Afterward, the number of fractures in each section was counted.

A tagged visual analog scale developed for keel bone deviations in laying hens by Jung et al. (2022) was used to rate the severity of deviations displayed on the X-ray pictures. The rating was conducted by a single observer who had completed the online training supplied by Jung et al. (2022) before.

**FIGURE 2:**
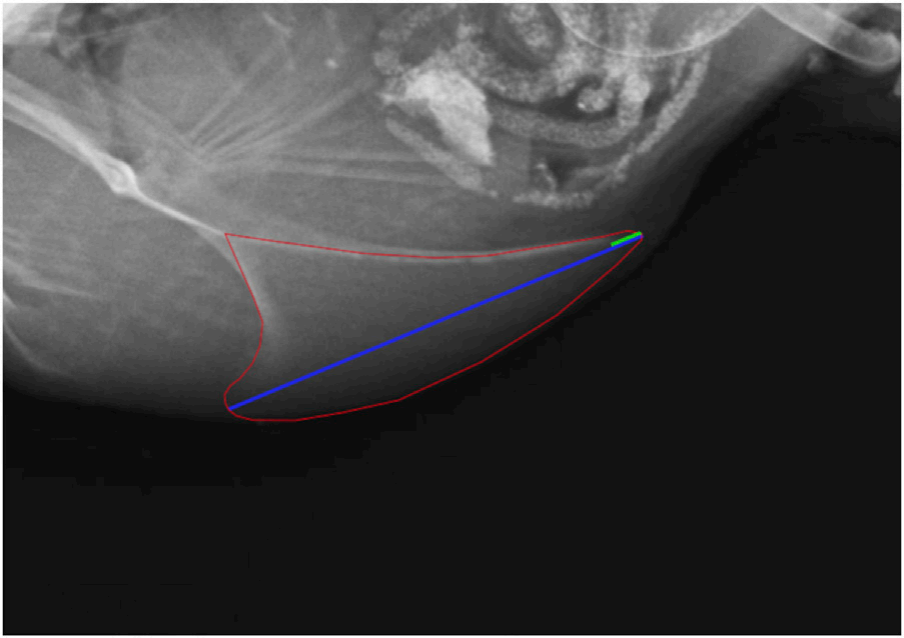
Measurement of the lateral surface area (red), the length (blue) and the cartilaginous portion of the keel bone (green).

Due to blurry images and tilting five radiographs had to be excluded from evaluation for radiographic density, lateral surface area, and length of the keel bone. For the measurement of the lateral surface area and length of the keel bone as well as the length of the cartilaginous part, the software Axio Vision 4.8 (Carl Zeiss Microscopy GmbH, Jena, Germany) was used. The lateral surface area was measured by encircling the keel bone, including the caudal cartilaginous part, and the length by drawing a straight line from the most cranio-ventral part to the most caudal part of the carina. The length of the cartilaginous part was also measured along that line (Figure 2). Area and lengths were then calculated with the help of a given scaling. For the measurement of the radiographic density, the aluminum step wedge provided the data to generate a calibration curve with a third polynomial function. An individual calibration curve was created for each radiograph. Then the area of the keel bone without the cartilaginous part was encircled and the software ImageJ (Version 1.52; Rasband, W; National Institute of Health, Bethesda, Maryland, United States) was used to transfer the mean gray value of the area into mm of aluminum equivalent as described by Eusemann et al. (2020). Further, to validate that the scaling of the X-ray pictures did not differ between the dates of examination, the width of the aluminum step wedge was measured and compared.

#### 2.3.4 Recording laying performance, egg weight, and eggshell weight

From the beginning of the experiment onwards, the number of eggs was counted at the same time every day at group level (Supplementary Material 2). If there were days on which the eggs were not collected, the number of eggs found on the following day was equally between the days. One day per week, the eggs of the day were weighed and their shell weight was determined (Supplementary Material 3). For this purpose, each egg was weighed on an electronic analysis scale Kern PCB 100-3 (Kern & Sohn GmbH, Balingen, Germany) and cut open with dissection scissors (Carl Roth GmbH &Co-KG, Karlsruhe, Germany) along the center to divide the egg into two-halves. The inner components were completely collected in a beaker (Carl Roth GmbH & Co. KG, Karlsruhe, Germany). The two-halves of the eggshell were numbered and placed in a paper egg tray with their inner surface towards the opened part of the tray. Afterward, the tray was placed in a compartment drier (Memmert UF750plus; Memmert GmbH u. Co. KG, Schwabach, Germany) for 24 h at 45°C to completely remove water containing components like yolk and egg white. Finally, the egg shells were removed from the egg tray using a pair of tweezers (Carl Roth GmbH & Co. KG, Schwabach, Germany) and weighed on an electronic analysis scale (Kern PCB 100-3; Kern & Sohn GmbH, Balingen, Germany).

#### 2.3.5 Maceration of the keel bones

For the maceration, the sternum was separated from the surrounding tissue using scissors (Wirtschaftsgenossenschaft deutscher Tierärzte eG, Gabsen, Germany) and a scalpel (Wirtschaftsgenossenschaft deutscher Tierärzte eG, Gabsen, Germany). It was then placed into a 1 L beaker (Carl Roth GmbH & Co.KG, Karlsruhe, Germany) and covered with a solution, which was made of 85% water, 10% Biozym SE (Spinnrad GmbH, Bad Segeberg, Germany), and 5% Biozym F (Spinnrad GmbH, Bad Segeberg, Germany). The beaker was then placed in a compartment drier (Memmert UF750plus; Memmert GmbH u. Co. KG, Schwabach, Germany) at 60 °C overnight (approximately 15–18 h). Afterward, the solution was poured through a strainer and the remaining tissue was removed from the bones. This procedure was then repeated one more time and finally the sternum was placed in a compartment drier at 35°C–45 °C overnight for drying. They were then stored in labeled containers until evaluation.

At the evaluation, two observers assessed the macerated keel bones for the absence or presence of keel bone deviation. The sensitivity was calculated by dividing the number of animals that showed keel bone deviation on both, X-ray image at 23 weeks of age and macerated keel bone by the total number of animals that showed deviation on their macerated keel bone. The specificity was determined by dividing the number of animals that showed neither a deviation on the X-ray image at 23 weeks of age nor on the macerated keel bone, by the number of animals without deviation on their macerated keel bone.

#### 2.3.6 Histological analysis

**FIGURE 3:**
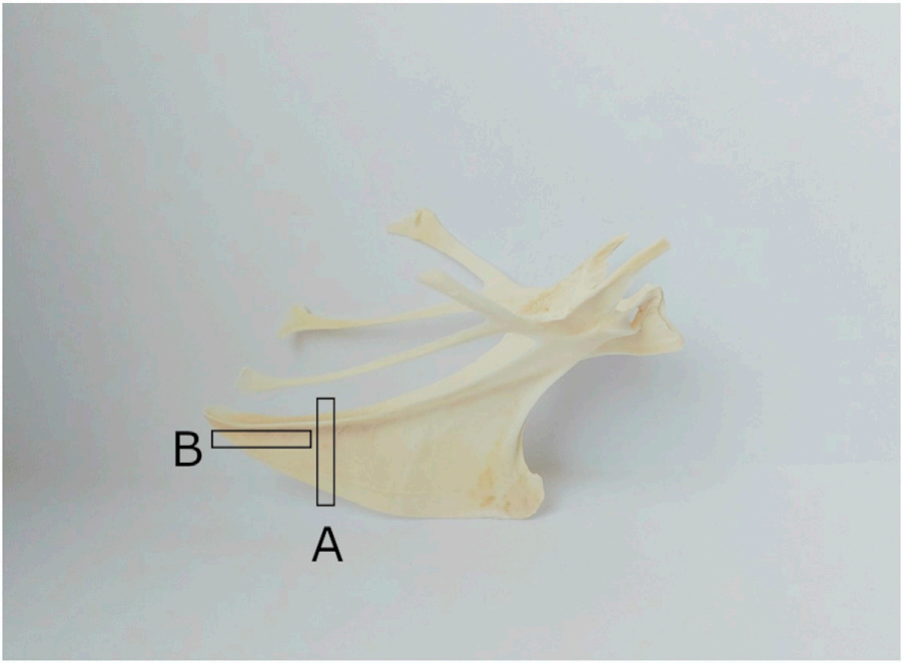
Cutting of the keel bone for transversal and horizontal sections (**(A)** for transversal sections, **(B)** for horizontal sections).

For the histological analysis of the quail keel bone, samples of ten 20-week-old female Japanese quails were used. These quails were not identical to the quails that were radiographed. Instead, they had been part of another study and we received their keel bones as part of an organ sharing initiative. After separation from the muscles, pieces for transversal and horizontal sections were cut from each keel bone. For the transversal section, a piece of 3–4 mm in thickness was cut from the caudal third of the keel bone. For the horizontal section, a piece of 3–4 mm in thickness was cut from the caudal part of the keel bone parallel to the corpus of the sternum (Figure 3). The pieces were then decalcified for 6 weeks in buffered ethylene-diamine tetra acetic acid (EDTA; Carl Roth GmbH + Co. KG, Karlsruhe, Germany). Afterward, they were washed in tap water, dehydrated through an ascending alcohol series, and embedded in paraffin wax (Surgipath Paraplast plus, Leica, Illinois, United States). Finally, the sections were cut at 5 μm with a rotary microtome (Microm HM 355 S, Thermo Fisher Scientific™ Inc., Waltham, Massachusetts, United States), mounted on a slide covered with poly-l-lysine (Thermo Fisher Scientific Inc., Waltham, Massachusetts, United States), and stained with hematoxylin and eosin (Hematoxylin solution acc. to Delafield, Carl Roth GmbH + Co. KG, Karlsruhe, Germany) In order to assess how the medullary bone is distributed in the keel bone of laying female quails, horizontal and longitudinal sections of the keel bone were examined at ×2.5 and ×20 magnification with a light microscope (Axio Imager 2, Carl Zeiss AG, Oberkochen, Germany).

#### 2.3.7 Statistical analysis

Statistical analysis was performed with RStudio 2023.03.0 and Excel (Version 1808; Microsoft, Redmond, Washington, United States).

The length of the cartilaginous part of the keel bone was calculated as the percentage of the total keel bone length. For the length of the cartilaginous part (log-transformed), body weight, radiographic density, keel bone length, keel bone lateral surface area, and the score of the deviation (log-transformed), mixed models with week of age as fixed effect were created using the nlme package and an ANOVA was performed. The animal was included as a random factor to take the repeated measures into account. The residuals were controlled for normal distribution by visual examination using q-q-plots and histograms. Post-hoc analysis was performed using Tukey’s HSD tests.

As there were animal losses throughout the study (see 2.2), the daily egg-laying rate was calculated by dividing the number of eggs by the number of hens. As quails are reported to enter full lay between the eighth and ninth week of age (Gerken and Mills, 1993), only the laying rates from week 10 to week 23 were considered in order to assess whether there were any changes in laying performance throughout the laying period. As conducted before, a mixed model with the week of age as fixed and the animal as random effect was created. The residuals were visually controlled for normal distribution and Tukey’s HSD tests were performed for post-hoc analysis.

The relative weight of the eggshell was calculated by dividing the weight of the eggshell by the weight of the whole egg. To assess changes during the laying period, a linear model with week of age as a fixed effect was created and an ANOVA was performed.

## 3 Results

### 3.1 Body weight

The body weight was significantly influenced by age (F_4,161_ = 13.31, *p* < 0.0001). There was a significant increase in body weight between weeks 8 and 10 (z = 4.53, *p* < 0.0001). From week 10 onwards, no significant changes in body weight could be detected. However, there was a trend of an increase (z = 2.71, *p* = 0.067) between weeks 19 and 23. For more detailed information, the weight data can be found in Supplementary Material 1.

### 3.2 Laying rate and eggshell weight

**FIGURE 4:**
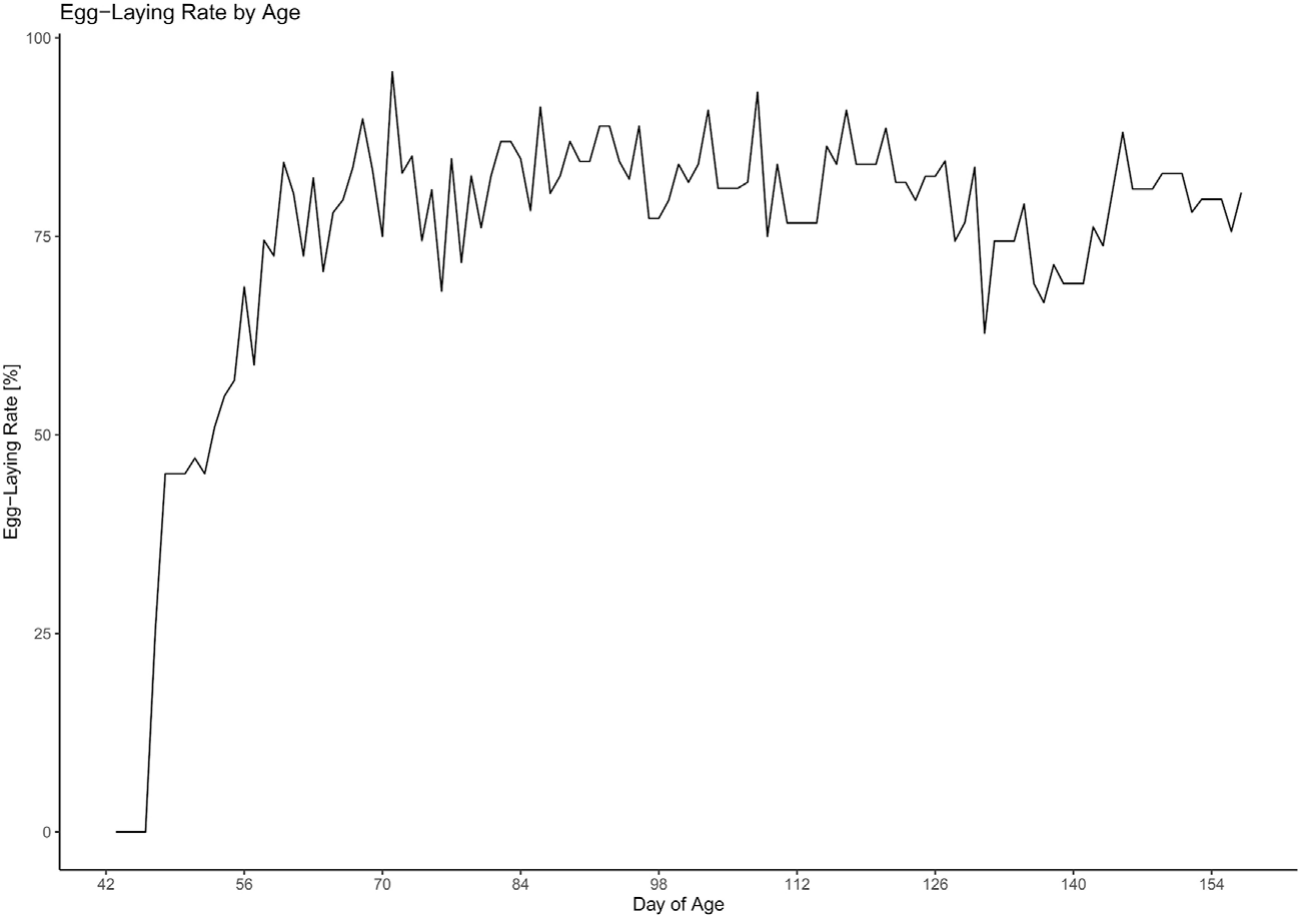
Development of the egg-laying rate throughout the experiment.

The daily egg-laying rate can be viewed in Figure 4. There were strong fluctuations between the days. The egg-laying rate first inclined in week 7 and then seemed to reach a plateau in week 10. Statistical analysis revealed that during the time between weeks 10 and 23 there were significant differences between the weeks (F_13_ = 2.95, *p* = 0.0015) with the egg-laying rate being significantly lower in week 20 compared to weeks 13 (z = −4.4, *p* = 0.003), 14 (z = −2.79, *p* = 0.0033), 15 (z = −4.11, *p* = 0.0086), 17 (z = −4.13, *p* = 0.008), and 18 (z = −4.04, *p* = 0.0111) and tending to be lower compared to weeks 11 (z = −3.6, *p* = 0.0509), 12 (z = −3,58, *p* = 0.0532), and 16 (z = −3.43, *p* = 0.0884).

There was no significant effect of week of age on the relative eggshell weight.

### 3.3 Fractures

**FIGURE 5:**
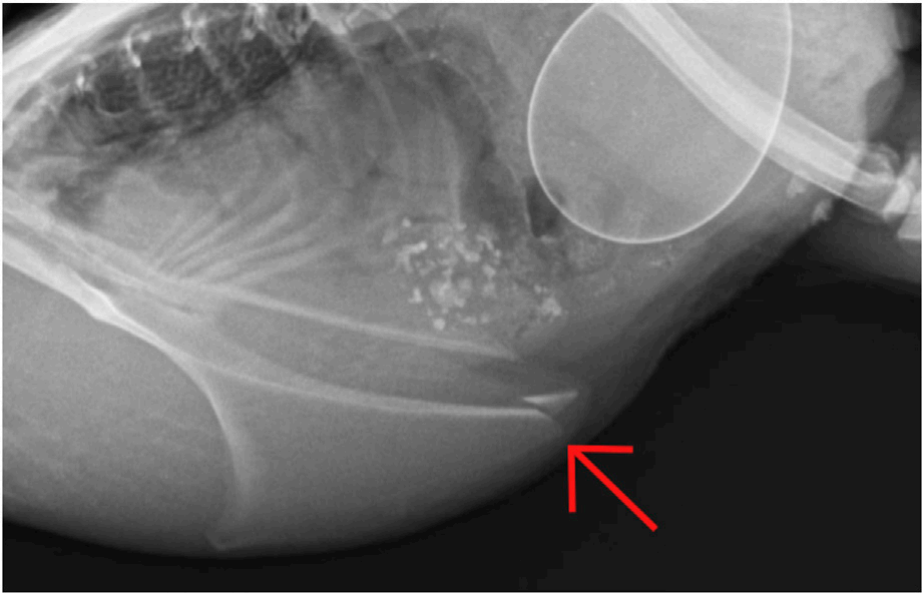
Keel bone fracture in section C (caudal third) at 15 weeks of age.

Throughout the experiment, fractures were seen in a total of three animals. There were only single fractures, no multiple fractures. In terms of location, two of the fractures were found in the caudal third (section C; Figure 5) and one in the cranial third (section A) of the keel bone. The fractures in section C were present in weeks 8 and 15, respectively. There was no callus formation visible at the subsequent dates of examination. The fracture in section A was first detected in week 19 with callus formation in week 23.

### 3.4 Deviations

In the X-ray pictures, deviations were scored with a maximum of 2 out of 10 which indicates minor deviation. The overall average score was 0.27 ± 0.42. Statistical analysis showed significant changes with age (F_4,161_ = 11.56, *p* < 0.0001): A significantly higher deviation score was seen from week 15 onwards compared to week 8 (z = 3.82, *p* = 0.0014) and in week 23 compared to week 10 (z = 4.33, *p* = 0.0001). Prevalence of deviation varied between 19.6% and 55.8% during the study when assessed with radiography while maceration revealed that 82.1% of the keel bones were deviated (Table 1). Compared to the outcome of the maceration, the sensitivity and specificity of the radiography for detecting keel bone deviation were 56.25% and 57.14%, respectively.

**TABLE 1:** Keel bone deviations: Severity scores and prevalence throughout the experiment.

	Week 8	Week 10	Week 15	Week 19	Week 23	*Post mortem* dissection
Ø Score of deviation	0.1 ± 0.32	0.2 ± 0.35	0.3 ± 0.43	0.3 ± 0.44	0.4 ± 0.55	-
Minimum score of deviation	0	0	0	0	0	-
Maximum score of deviation	2	2	2	2	2	-
Prevalence [%]	19.6	41.3	55.8	53.7	48.6	82.1

### 3.5 Lateral surface area, length, and ossification of the keel bone

Both, the length (F_4,161_ = 85.27, *p* < 0.0001) and the lateral surface area (F_4,161_ = 69.83, *p* < 0.0001) of the keel bone were significantly influenced by age.

**FIGURE 6:**
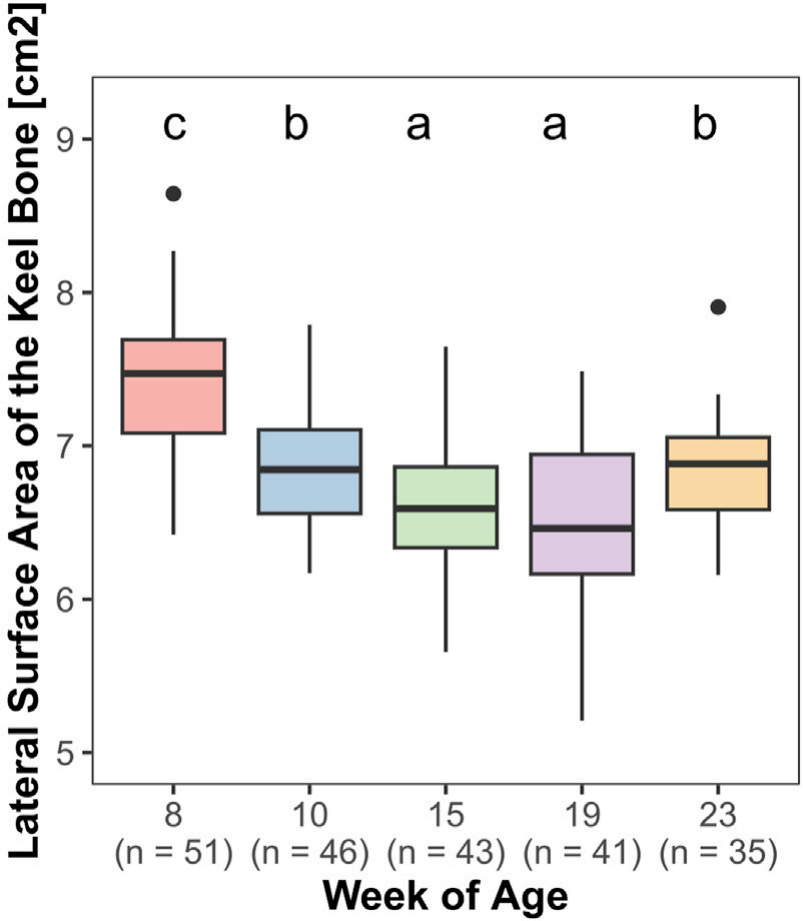
Lateral surface area of the keel bone.

**FIGURE 7:**
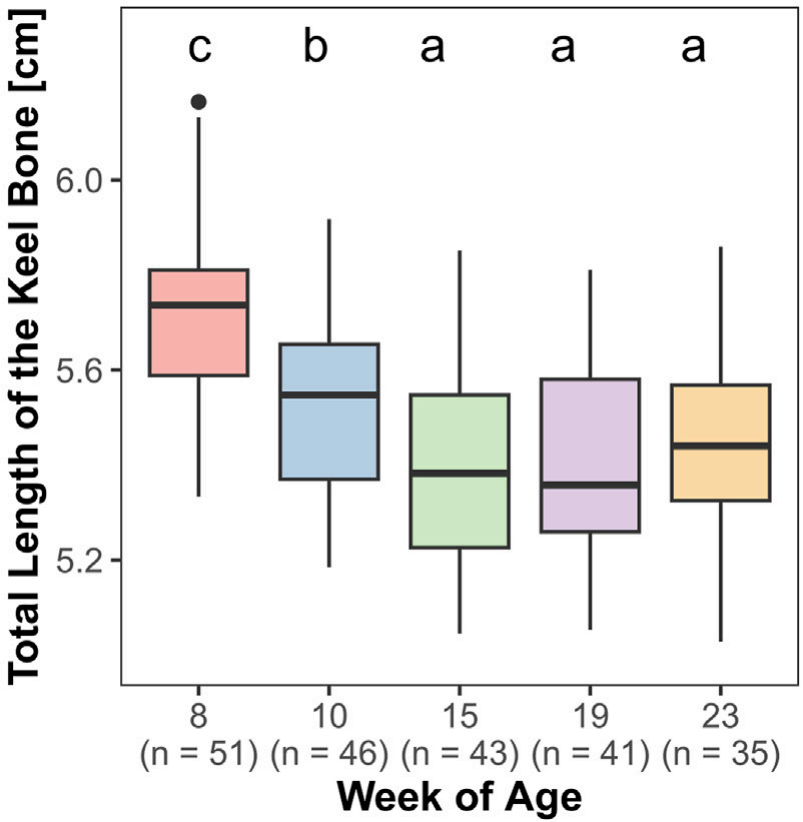
Total length of the keel bone.

The lateral surface area of the keel bone gradually decreased between the eighth and the 10th (z = −9.4, *p* < 0.0001) and the 10th and the 15th week of age (z = −4.58, *p* < 0.0001), then increased between the 19th and 23rd week of age (z = 5.3, *p* < 0.0001) (Figure 6). Likewise, the length of the keel bone decreased between weeks 8 and 10 (z = −9.47, *p* < 0.0001) and weeks 10 and 15 (z = −6.3, *p* < 0.0001) and then leveled off (Figure 7).

The mean of the absolute and relative length of the cartilaginous part of the keel bone as well as the maximum and minimum and the percentage of animals whose keel bones were fully ossified can be viewed in Table 2. Statistical analysis showed a significant effect of age on the relative length of the cartilaginous part (F_4,161_ = 280.73, *p* < 0.0001) with a decrease between weeks 8 and 10 (z = −23.35, *p* < 0.0001) and weeks 10 and 15 (z = −3.57, *p* = 0.0035). From week 19 onwards, no cartilage component could be identified anymore.

**TABLE 2:** Progress of ossification: Length of the cartilaginous part and percentage of animals with fully ossified keel bones.

	Week 8	Week 10	Week 15	Week 19	Week 23
Ø Length of the cartilaginous part of the keel bone [cm]	0.53 ± 0.18	0.08 ± 0.11	0.01 ± 0.03	0	0
Ø Relative length of the cartilaginous part of the complete keel bone [%]	9.3 ± 3.2	1.4 ± 2	0.1 ± 0.6	0	0
Minimum length of the cartilaginous part of the keel bone [cm]	0	0	0	0	0
Maximum length of the cartilaginous part of the keel bone [cm]	0.89	0.36	0.17	0	0
Percentage of animals with fully ossified keel bones [%]	5.9	63	95.3	100	100

### 3.6 Radiographic density of the keel bone

**FIGURE 8:**
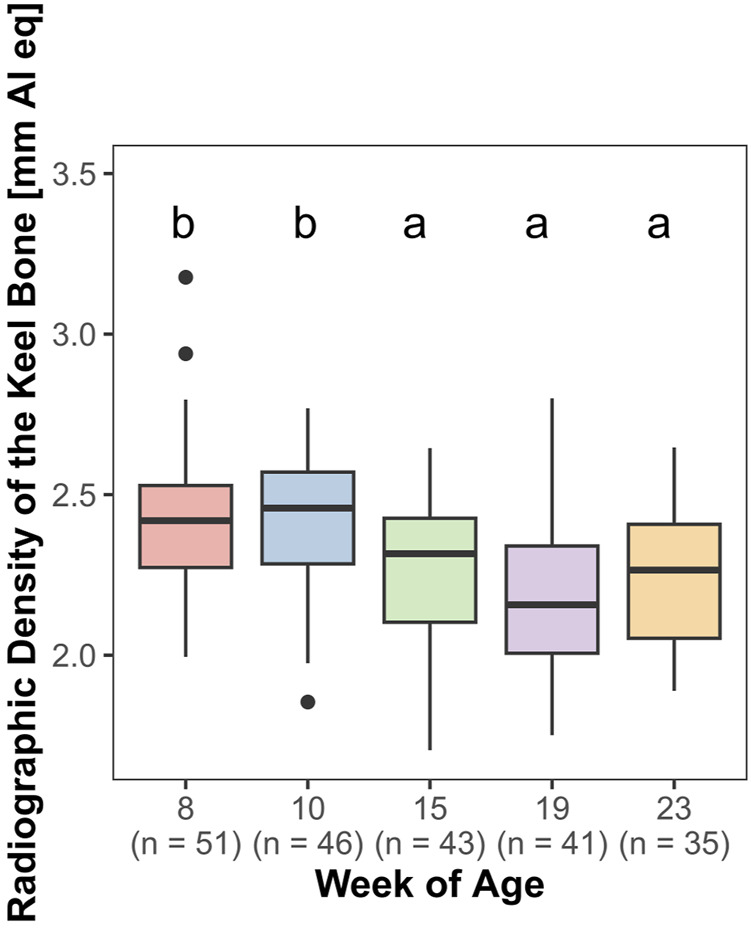
Radiographic density of the keel bone.

The radiographic density of the keel bone was significantly influenced by age (F_4,161_ = 18.84, *p* < 0.0001). There was a significant decrease between weeks 10 and 15 (z = −4.76, *p* < 0.0001). In contrast, there were no significant changes in radiographic density between weeks 8 and 10 and weeks 15–23 (Figure 8).

### 3.7 Histological analysis

**FIGURE 9:**
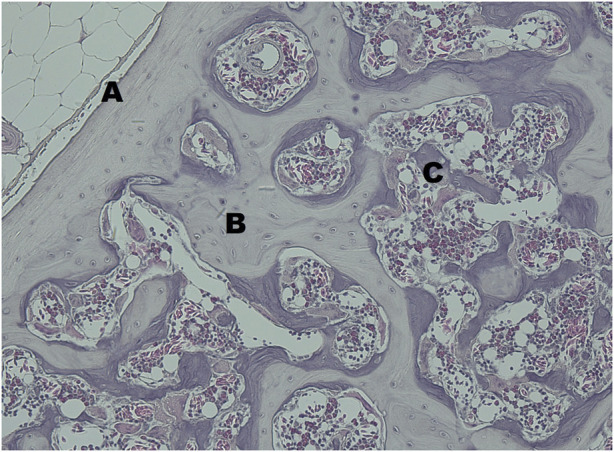
The transversal section of the keel bone of a female quail at 20 Weeks of age stained with haematoxylin and eosin at ×20 magnification. **(A)** cortical bone, **(B)** trabecular bone in bright violet, **(C)** medullary bone, in dark violet.

All examined keel bones contained basophile short spicules, which were identified as medullary bone (Figure 9). They were detectable in both, transversal and horizontal sections, and were located on the endosteal surface of the cancellous, i.e., trabecular bone.

## 4 Discussion

Our results clearly show that both, keel bone fractures and deviations occur in Japanese quail. In addition, we observed changes in the radiographic density, length, and lateral surface area of the keel bone during the laying period.

### 4.1 Laying performance, eggshell weight, and body weight

The rather strong variation of the egg-laying rate between the individual days is probably due to the fact that quails were not provided with nests and laid their eggs in the sand. Therefore, it cannot be guaranteed that all eggs were found on the same day. It has to be taken into account, that the number of eggs found in the sand may differ from the number of eggs that were actually laid. Between weeks 10 and 23, there was a significantly lower laying rate in week 20 than in other weeks. Even if no signs of illness or increased mortality were reported, it cannot be fully excluded that this reduction in performance was caused by stress or other external factors. In addition, we can not completely eliminate that fewer eggs were found in the sand this week due to management errors. However, another explanation for the decrease in laying performance could be that more animals showed physiological clutch pauses during this period (Aggrey et al., 1993).

Analysis of the relative weight of the eggshell showed no significant effect of age. This is in contrast to findings in laying hens (Silversides and Scott, 2001) where the relative weight of the eggshell declines in progress of the laying period. A possible explanation for the different findings could be the shorter production and observation period in the present study.

The significant increase in body weight between weeks 8 and 10 suggests that the quails are still growing at that age and therefore gain body mass. This is supported by the fact that the ossification of the keel bone is not completed in the majority of animals at that age. Another reason for the increase could be the incline in laying activity. When hens enter lay, this is accompanied by an increase in the weight of the reproductive organs (Kwakkel et al., 1995).

### 4.2 Keel bone fractures and ossification

Overall, keel bone fractures were detected in three out of the 51 animals, which equals 5.9%. Two out of these three fractures were located in the caudal third of the keel. This matches the findings of Baur et al. (2020) in laying hens, where 77% of keel bone fractures occurred in the caudal third of the keel. In contrast to laying hens that frequently show multiple fractures (Thøfner et al., 2021), only single fractures were found in Japanese quail in the present study.

Even if these numbers seem to be relatively low compared to the number of fractures in laying hens, where keel bone fractures can affect up to 100% of the animals in a flock (Thøfner et al., 2020), it has to be taken into account that the quails were observed for a shorter period and more fractures could have occurred if they had been observed for a longer period. Depending on the author, production periods for laying quails last from 24 up to 40 weeks (Lukanov et al., 2018; Damme et al., 2021). Also, it has to be taken into account that due to the animal losses not all 51 quails were observed until the end of the study.

There may also be a reduced risk for traumatic fractures through collisions and falls since quails do not roost. This would be consistent with the relationship between accessible height and keel bone fractures in laying hens described by Wilkins et al. (2011) and laying hens in floor housing showing fewer keel bone fractures than those kept in aviaries (Jung et al., 2019).

However, the lower number of keel bone fractures could also be explained by differences in the calcium metabolism between quail and laying hens. Even if quails and laying hens show a similarly high laying performance in terms of egg number, the absolute and relative content of calcium is higher in chicken eggshells (Navarro and Murillo, 1976; Ajala et al., 2018; Kalbarczyk et al., 2022). Furthermore, in contrast to chicken, domesticated Japanese quail lay their eggs in the afternoon (Yamashina, 1961). Andrade et al. (2023) observed that most parts of quail eggshell are secreted during the day and, thus, suggest that the calcium source from the medullary bone has less importance in quail than in commercial laying hens, which lay most eggs in the morning and therefore their eggshell is formed at night, when no calcium is absorbed from food (van de Velde et al., 1984; Samiullah et al., 2016).

Another factor that could contribute to a lower prevalence of keel bone fractures is the earlier completion of ossification of the keel bone in Japanese quail as observed in our study. Buckner et al. (1949) showed that in New Hampshire chicken, at 20 weeks of age (i.e., at the onset of lay) 50% of the keel bone was still cartilage and complete ossification of the keel was not reached before 35–40 weeks of age. In contrast, the average cartilaginous part of the quail keel at 8 weeks of age, when the quails started to lay, was 9.3% and all keels were fully ossified at 19 weeks of age. Also, the lack of significant changes in body weight from the 10th week onwards suggests that the quails are fully grown at that point. In laying hens, fractures of the caudal part of the keel bone often show an appearance similar to greenstick fractures (Thøfner et al., 2020). In humans, greenstick fractures are bending fractures that occur in the calcified cartilage of pediatric patients before complete ossification (Kittleson and Whitehouse, 1966). As the ossification of the keel bone is further progressed at the onset of lay in Japanese quail, they may be less likely to experience greenstick fractures.

The presence of keel bone fractures in Japanese quail proves that this condition is not restricted to chicken but also affects quail and could affect other bird species (e.g., ducks, geese) that are used for egg production as well if they show an increased laying rate compared to the wild type. As in chickens, high laying performance may contribute to the appearance of keel bone fractures in female Japanese quail, too. Our results also point out the ossification of the keel at the onset of lay as a key factor for the development of caudal keel bone fractures. As laying hens with keel bone fractures experience pain (Nasr et al., 2012; 2013), quails with keel bone fractures are most likely to experience pain as well. Thus, keel bone fractures in laying quail should be kept in mind as a potential animal welfare problem and included in welfare protocols for this species.

### 4.3 Keel bone deviations

When assessed with radiography, 60.8% of the 51 quails showed deviations at one or several examination dates. The percentage of deviated keel bones that were discovered through maceration at the end of the experiment was 82.1%. These results are similar to the prevalence of deviations found in laying hens (Eusemann et al., 2018a; Jung et al., 2022). Due to the rather low sensitivity and specificity of radiography in this study, X-ray examination does not seem to be an adequate tool for detecting keel bone deviations in Japanese quail. In laying hens, it has been shown to be a useful tool for assessing keel bone deviations by some authors while others found a rather low sensitivity of 60%, too (Tracy et al., 2019). Keel bone deviations may be difficult to detect with radiography in Japanese quail as they are less severe than the ones found in laying hens. The Japanese quails in the present study showed an average score of 0.27 with a maximum of 2 out of 10 while laying hens were scored with an average score of 2.48 and a maximum of 10 out of 10 (Jung et al., 2022). During perching, high peak forces impact the keel bone of laying hens (Pickel et al., 2011). As quails do not use perches and, thus, do not experience the same kind of peak force on their keel bone, they are likely to develop fewer and less severe sagittal deviations than laying hens. Since only lateral X-ray pictures were taken, sagittal deviations were easier to detect than transversal ones. However, it was not possible to evaluate postero-anterior radiographs in former studies due to the small ventral surface of the keel bone and projections with other body parts (Eusemann et al., 2018a). Thus, no postero-anterior radiographs were taken in the present study. As the sagittal deviations in the present study were rather slight and barely exceeded a straight line, it was not practicable to measure the proportion of deviated keel bone area (POD) in the X-ray images as it was done by Eusemann et al. (2018a).

Our results show that the prevalence of keel bone deviations in Japanese quail is comparable to that in laying hens, even if they are less severe. A possible common factor is rearing in the absence of ultraviolet-B (UV-B) radiation. Both, quails and laying hens, are usually reared inside a barn without access to the full spectrum of sunlight. Doyle (1925) already described a bend of the keel bone as a symptom of rickets in mature chickens that occurs when young chickens are housed inside. UV-B plays a major role in the synthesis of Vitamin D3. Vitamin D3 activates the calcium-binding proteins in the intestine (Bar et al., 1976). Although dietary Vitamin D3 is supplemented, UV-B radiation has been shown to have a better effect on the bone health of young chicks compared to dietary supplementation of Vitamin D3 (Edwards, 2003). The high prevalence of keel bone deviations in both, laying hens and Japanese quail, emphasizes the need of further investigation on the cause and impact of deviations on the animals. Furthermore, our study indicates that radiography is not sensitive enough to detect all keel bone deviations in quails and should therefore be supplemented with another technique such as palpation or maceration.

### 4.4 Radiographic density, lateral surface area, and length of the keel bone

There was a significant decrease in radiographic density between the 10th and 15th weeks of age. At this time, the quails reached their laying maximum. Therefore both, the loss of bone mass due to the high calcium demand for eggshell formation and a shift in bone composition in favor of medullary bone are conceivable. In a study by Eusemann et al. (2018b), radiographic density initially increased until week 33 but then stayed constant throughout the study in untreated laying hens. This is in contrast to our study. However, non-egg-laying hens showed higher radiographic density values compared to control hens (Eusemann et al., 2020), supporting the hypothesis that radiographic density may be negatively influenced by eggshell formation. In humeri, the bone mineral density has been shown to be linked to the breaking strength of the bone (Fleming et al., 2000). Toscano et al. (2018) showed that a greater bone mineral density provides a protective effect at low collision energies, but increases the possibility of severe fractures at high collision energies. It should be kept in mind that the *in vivo* assessment of radiographic density may be influenced by other factors such as feathers, skin, and muscles and not by the actual density of the bone alone. However, these factors were approximately the same in our study at all times and in all animals. Therefore, in our opinion, it cannot be assumed that this had an effect on the results.

Both, the length and the lateral surface area of the keel bone, decreased between the 8th and 19th week of age and then increased again until the 23rd week of age. It is possible that there is a loss of bone substance due to the calcium demand for eggshell formation. A decrease in keel bone area throughout the laying period has been noticed in laying hens before and was the reason for Eusemann et al. (2018a) to establish the “Proportion of deviated keel bone area, POD” to observe the area of deviation in proportion to the overall area of the keel bone.

The increase in keel bone length and area between weeks 19 and 23 could indicate a recovery of the bone substance. A decrease in new fractures for older birds, which could indicate a recovery of bone substance, too, has also been reported for laying hens (Jung et al., 2019; Baur et al., 2020).

The recovery of bone substance could also be due to the decrease in laying performance in week 20, which might stand for laying pauses. Further studies would have to show if this possible recovery is reproducible.

### 4.5 Conclusion

In our study Japanese quails suffered from keel bone damage. Furthermore, we could observe changes in keel bone features such as length, radiographic density, and lateral surface area, which could indicate loss of bone substance/mineralization throughout the laying period. In our opinion, further research is required to gain more information about the extent of keel bone damage in layer quails and the effect of their high laying performance on bone quality. It should also be investigated in further studies whether the damage to the sternum has the same aetiology as in laying hens and whether there are other parallels in this clinical picture.

## Data Availability

The original contributions presented in the study are included in the article/Supplementary Material, further inquiries can be directed to the corresponding author.
